# Structural Engineering and Functionalization of Carbon-Based Anodes for Sodium-Ion Batteries: From Biomass to Composites

**DOI:** 10.3390/molecules31050843

**Published:** 2026-03-03

**Authors:** Bushra Iqbal, Adam Moyseowicz

**Affiliations:** Department of Process Engineering and Technology of Polymer and Carbon Materials, Faculty of Chemistry, Wrocław University of Science and Technology, Gdańska 7/9, 50-344 Wrocław, Poland; bushra.iqbal@pwr.edu.pl

**Keywords:** Na-ion batteries, sustainable materials, graphene-based materials, electrochemical energy storage systems

## Abstract

Sodium-ion batteries (SIBs) are becoming more popular as a sustainable and affordable alternative to lithium-ion batteries for electric energy storage. One of the key challenges of SIB development lies in the cell components, including anode material capable of reversible hosting of Na^+^ ions. Carbon-based materials are still the best choice for this purpose because they can be modified easily and produced in larger quantities, while accommodating a large amount of stored sodium. This review provides an overview of hard carbon (HC)- and reduced graphene oxide (rGO)-based anode materials, from precursors made from biomass and polymers to structurally engineered graphene derivatives and carbon–transition metal composites. This review focuses on how the synthesis protocols, carbon structure properties, porosity, surface functionalization, and introduction of the inorganic components affect the sodium storage mechanism and performance. The review provides insights into rational material design strategies and underlines key challenges in the pursuit of scalable, high-efficiency SIB anodes.

## 1. Introduction

The significant efforts to transition from finite fossil fuels to sustainable energy sources led to the establishment of an electrified economy, wherein electricity serves as the predominant energy source for all industrial and social endeavors. Nevertheless, the inherent unpredictability of renewable energy sources, such as wind and solar power, necessitates the development of systems for electricity storage. Managing the surges and deficit of electric power created by renewable energy sources and the transportation sector is particularly vital in the electrical grid. The utilization of lithium-ion batteries (LIBs) has been prominent in the field of battery technology and is acknowledged as a pivotal sector in the forthcoming decarbonized society. However, despite the benefits, lithium-ion batteries include notable drawbacks that pose a danger to their future viability and technical dominance. The primary concerns pertain to the environmental ramifications and the exploitation of crucial and finite resources, including cobalt, lithium, and natural graphite [1]. In recent years, there has been a growing focus on environmental and technical safety problems in many applications, mostly driven by the growing presence of electric vehicles (EVs) in the broader market. Scientists are investigating various battery compositions to overcome the limitations of current lithium-ion batteries [2]. These include several combinations of anodes and cathodes, such as sodium-ion, magnesium, lithium–sulfur (Li-S), and lithium–air. They also comprise a range of technologies, including redox flow batteries. 

In order to be used as replacements in industries like automotive and stationary applications, post-LIBs must possess similar characteristics to LIBs. Sodium-ion batteries (SIBs) have emerged as the most sophisticated among post-LIB improvements, and numerous start-ups are actively striving to bring their innovations to the commercial market. Sodium-ion batteries have a fundamental resemblance to LIBs due to their substantial overlap in electrochemical characteristics. The technology in question is a plug-and-play system that necessitates only minimal technological modifications for the purpose of transitioning manufacturing lines or fabrication of battery packs.

SIBs offer several strategic advantages over LIBs, primarily due to the natural abundance and low cost of sodium resources, which significantly reduce material and production expenses. Unlike LIBs, sodium-based devices exhibit superior low-temperature performance because Na-based electrolytes maintain higher ionic conductivity under subzero conditions [3]. The cathode materials of SIBs exhibit higher thermal stability, eliminating the risk of lithium plating and enhancing their intrinsic safety. Sodium-based systems can be assembled with larger particle sizes and simpler manufacturing routes. These characteristics make SIBs particularly attractive for grid-scale energy storage, renewable-energy buffering, and other stationary applications where cost, safety, and sustainability outweigh the need for maximum energy density [4].

The primary benefits of SIBs are rooted in their improved safety characteristics, which result in a notable decrease in the likelihood of explosions and fires. Additionally, SIBs employ cost-efficient and easily accessible materials, such as aluminum in place of copper for the current collector, sodium in place of lithium in the cathode active material and electrolyte salt, and cathode materials that are free from cobalt. The pursuit of revolutionary battery technologies is primarily driven by economic and environmental considerations, positioning SIBs as a formidable candidate for future battery solutions.

## 2. Carbon Materials for Sustainable Na-Ion Battery Anodes

Before introducing specific carbon precursors, it is essential to outline the fundamental performance requirements for SIB anodes. High reversible capacity, suitable operating potential, long cycle life, high initial Coulombic efficiency, and stable Na^+^ diffusion kinetics are crucial for achieving competitive SIB performance. Because Na^+^ has a larger ionic radius than Li^+^, SIB anodes must also tolerate greater structural strain during cycling. Based on their sodium storage mechanisms, SIB anodes are generally categorized into intercalation-type, conversion-type, alloying-type, and alloying–conversion hybrid materials, each offering distinct advantages and limitations [5]. Within this broader landscape, hard carbon has emerged as the most practical and industrially relevant anode material due to its low operating potential, structural disorder, tunable porosity, and excellent cycling stability. The anode materials used for SIBs in the early stages of development were predominantly hard carbon, and their applications remained challenging [6]. Although biomass-derived hard carbon has attracted significant attention due to its sustainability and low cost, its intrinsic structural complexity presents challenges. Biomass precursors contain varying proportions of cellulose, hemicellulose, lignin, and inorganic impurities, which strongly influence carbonization behavior and microstructure evolution. As a result, parameters such as pore size distribution, defect density, interlayer spacing, and oxygen content can vary between batches, leading to limited reproducibility and inconsistent electrochemical performance. Zhang et al. introduced the controlled pore engineering strategies; regulated closed-pore formations provide a more precise approach to adjust pore configuration, interlayer spacing, and defect density, enabling more reproducible electrochemical performance [7,8]. Experimental and theoretical studies have widely investigated several carbon-based materials as the electrode material for energy storage and conversion devices, including those related to fuel cells, supercapacitors (SCs), and LIBs, aiming at improving the specific capacity, cyclic efficiency, lifetime, power, and energy density of these energy storage devices. In the 1980s, sodium-ions were electrochemically inserted into carbon-based materials. Hard carbon electrode materials have a special combination of chemical and physical properties, including high abundance, excellent corrosion resistance, moderate conductivity, relatively high surface area, high initial Coulombic efficiency (ICE), stable cyclic capacity, and low cost. Researchers believe that they can improve the performance of energy storage device electrodes [9]. Therefore, we can still carry out rational design of high-performance hard carbon to some degree, and these attempts will also help to refine the sodium storage mechanism in return. Continuous efforts have led to the development of high-performance hard carbon anodes [10,11,12,13,14]. In addition to these advantages, hard carbon possesses a unique defect-rich and turbostratic structure that is fundamentally different from graphite. Hard carbon typically exhibits enlarged interlayer spacing (0.37–0.45 nm), abundant edge defects, disordered graphene layers, and closed or semi-closed nanopores. When the defect density and porosity are optimized, these intrinsic structural characteristics allow hard carbon to deliver a high reversible capacity. In contrast, graphite has a highly ordered layered structure with a small interlayer spacing of ~0.334 nm, which is insufficient to accommodate the larger Na^+^ [15]. As a result, graphite cannot form stable Na intercalation compounds and delivers negligible capacity in sodium ion systems [16]. This fundamental limitation has been confirmed by both experimental and theoretical studies, establishing hard carbon, not graphite as the most suitable carbonaceous anode for SIBs. Hard carbon anodes with controlled defect density and tailored porosity are widely reported to deliver reversible capacities primarily in the ≈200–400 mAh g^−1^ range with good cycling stability [17]. Xiao et al. [13] obtained a hard carbon anode with a low defect and low porosity, which delivered a high reversible capacity of 361 mAh g^−1^ and an excellent capacity retention of 93.4% after 100 cycles. Lu’s group [18] utilized electrospinning to synthesize a phosphorus-functionalized hard carbon anode which exhibited 393.4 mAh g^−1^ with the capacity retention up to 98.2% over 100 cycles. Similarly, heteroatom engineering through sulfur doping has been shown to further enhance sodium storage, with sulfur-rich hard carbon delivering reversible capacities as high as ~770 mAh g^−1^, attributed to additional redox-active sites and defect-mediated Na^+^ storage beyond conventional interlayer insertion [19]. Adjusting the micro-structure of hard carbon plays an important role in the rational design of hard carbon materials. In general, changing the precursors [10,20,21,22,23], optimizing the pyrolysis process and doping heteroatoms are the most common and effective methods to modify the microstructure. It is worth noting that heteroatom dopants such as sulfur, phosphorus, and boron, as well as co-doping systems (e.g., N-S, N-P), also influence active-site chemistry, electronic conductivity, and Na^+^ diffusion, thereby offering additional pathways for tuning the structural and electrochemical properties of hard carbon anodes [24,25]. The hard carbon precursors are mainly derived from a variety of organics or biomass [20,26,27], such as tea leaves [23], cellulose [21], cotton [28], and the different precursors usually exhibit different chemical components and microstructures and consequently result in hard carbon with different nano- and micro-structures. The cost of raw materials and yield should also be taken into consideration. Li et al. [29] obtained a HC anode from the cheap anthracite in an environmentally friendly way and the yield is as high as 90%, which is now being industrialized for its low cost. The pyrolysis process is also important for tuning the microstructure. By modifying the pyrolysis process, Zhao et al. [30] developed a hard carbon with a specific capacity of ∼400 mAh g^−1^, and 85% of its capacity was provided by the plateau part. It is also reported that the higher pyrolysis temperature may also lead to a smaller Brunauer–Emmett–Teller (BET) surface area and consequently a higher ICE [10,30,31,32].

**Figure 1:**
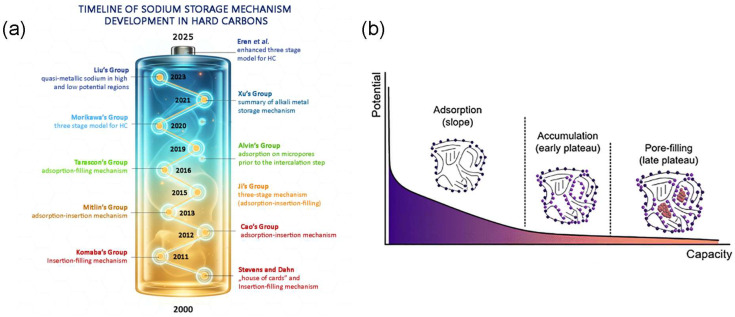
(**a**) Timeline of the key developments in the sodium storage mechanism occurring in the hard carbon anodes. Battery shape outline created using Google Nano Banana model (accessed January 2025), with further modifications by the authors in Gimp 3.0 software. All content was reviewed for accuracy. (**b**) The enhanced three stage mechanism of sodium storage in hard carbons. Reproduced from Ref. [41]. Copyright 2025 Royal Society of Chemistry. Reprinted with permission under Creative Commons Attribution license [CC BY 3.0].

The sodium storage mechanism in hard carbon (HC) remains under active scientific debate, primarily because HC is a structurally heterogeneous material in which multiple storage processes can coexist. Consequently, the dominant sodium storage mechanism depends strongly on the microstructural characteristics of the carbon, including defect density, interlayer spacing, and pore structure. The evolution of the proposed mechanisms is summarized in Figure 1a and shows that our understanding of phenomena in Na-ion batteries should therefore be interpreted not as mutually exclusive models, but as different manifestations of sodium storage governed by specific structural features of the carbon matrix [33,34]. The first systematic description was proposed by Stevens and Dahn in 2000, who introduced the “insertion–adsorption” mechanism based on glucose-derived hard carbon. In this model, graphitic layers exhibit a turbostratic arrangement resembling loosely stacked cards, where partially aligned graphene layers exist alongside disordered regions, creating micropores between layers that enable Na^+^ storage [35]. Subsequent studies, however, revealed inconsistencies with this interpretation, especially regarding the voltage-dependent storage behavior. Additional experimental evidence demonstrated that the sloping region of the galvanostatic charge–discharge (GCD) profile is primarily associated with Na^+^ adsorption at defect sites, edges, and surface functional groups, whereas processes occurring at lower potentials are related to storage within the carbon bulk. Based on the data derived from the X-ray diffraction (XRD), Cao et al. proposed the “adsorption–intercalation” mechanism, suggesting that Na^+^ ions are initially adsorbed at defective or disordered sites in the sloping region, followed by insertion into expanded graphitic domains in the plateau region, with an effective interlayer spacing (d002) of around 0.37 nm [36]. Bommier et al. proposed a “three-stage” model consisting of adsorption, insertion, and pore filling processes, in which sodium ions are first adsorbed at defects, then partially inserted into pseudo-graphitic layers, and finally stored within closed nanopores at low potentials [37]. Similar interpretations were later discussed by Zhang et al. [38]. Since 2020, the experimental and theoretical studies converged toward a unified interpretation in which sodium storage in hard carbon arises from three primary processes: (1) adsorption at surfaces, defects, and functional groups; (2) insertion into disordered or pseudo-graphitic interlayers; and (3) filling of closed nanopores or voids within the carbon structure [39,40]; however, recent operando investigations suggest that intercalation phenomena may appear negligible in most highly disordered HCs.

Operando SAXS, WAXS, and Raman studies reported by Eren et al. [41] have substantially refined this understanding and demonstrated that classical sodium intercalation into graphitic layers is negligible in most disordered hard carbons, as evidenced by the absence of significant lattice expansion or reversible structural changes during cycling. Instead, sodium storage follows an adsorption–accumulation–filling sequence, in which (1) a fast capacitive adsorption process dominates the sloping region, followed by a transition stage in the early plateau involving (2) faradaic accumulation of quasi-metallic sodium at micropore surfaces, and finally (3) multilayer-like clustering and pore filling in the late plateau region (Figure 1b). This operando interpretation helps connect the large plateau capacity with the minimal structural evolution observed during cycling. These sodium storage processes correspond to the characteristic slope region (>0.1 V) and plateau region (<0.1 V) of the galvanostatic charge–discharge (GCD) profile [42].

Understanding the correlation between the carbon microstructure and electrochemical response is therefore essential for the rational design of hard carbon anodes with optimized energy and power performance. Table 1 summarizes the major sodium storage models proposed for hard carbon over the years, emphasizing that mechanistic models should be viewed as structure-dependent limits within a unified framework rather than strictly separate categories.

### 2.1. Biomass-Derived Hard Carbons for Na-Ion Battery Anodes

**Figure 2:**
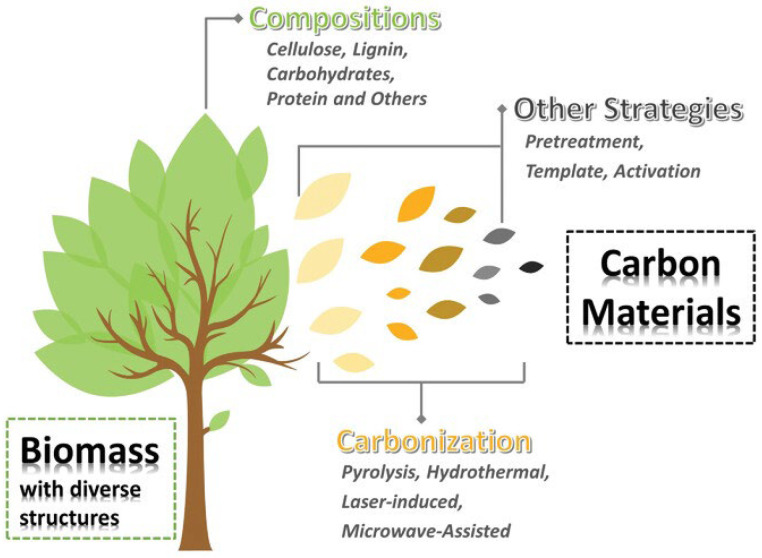
Conversion of biomass to carbon materials. The primary factors: Compositions, carbonization processes, and additional processing strategies. Reproduced from Ref. [43] with permission. Copyright 2021 Wiley GmbH.

Biomass-derived carbon materials (BCMs) are facing the most successful moment because of their versatile properties and wide potential applications [42]. Traditionally, biomass has been classified according to its origin into plant-derived and animal-derived types, which can be further categorized based on chemical composition—such as cellulose-based, lignin-based, carbohydrate-based, or chitin-based—or accordingly to the major component of biomass, which undergoes transformation into a carbonaceous structure in BCMs [43]. Figure 2 illustrates the main aspects that should be considered for the transformation process of biomass to carbon materials. Choosing the right biomass precursors is the key step for synthesizing BCMs with the desired structures and morphologies [44].

Plant-derived biomass mainly consists of cellulose, hemicellulose, and lignin, followed by organic extracts such as sugar, starch, protein, and inorganic minerals, etc. [45]. The proportion of cellulose, hemicellulose and lignin is different in various biomass resources [46]. The utilization of an inexpensive biomass resource as the precursor material for the production of hard carbons via a relatively innocuous process can offer an ample supply of low cost and good quality hard carbons for high areal and specific capacity anodes for SIB systems with an added benefit of enhancing environmental sustainability [47]. Structural features such as heteroatom content, pore architecture, and carbon skeleton regularity play a critical role in sodium storage behavior, providing essential guidance for the rational design of high-performance BCMs. Biomass-derived carbon owns a unique surface chemistry, crystalline structure, tuneable porous structure, and morphology [48,49,50,51,52,53], which is highly recommended for SIB anodes. Hence, it is a win-win situation to use biomass-derived carbon as the anode material for SIBs [42]. Therefore, the use of biomass-derived carbon as the anode material has emerged as an important valorization pathway for biomass energy.

**Figure 3:**
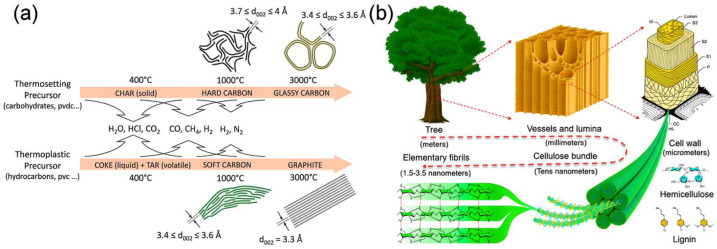
(**a**) Schematic representation of the typical microstructures of the carbons obtained at 1000 and 3000 °C. Reproduced from Ref. [54] with permission. Copyright 2018 Wiley GmbH. (**b**) Schematic illustration of the natural structure of wood, and the composition of the cell wall. Reproduced from Ref. [56] with permission. Copyright 2019 Wiley GmbH.

At high temperatures (1000–3000 °C), as seen in Figure 3a, BCMs display a large number of nanovoids and randomly oriented graphene layers [54], which is attributed to the structure being made up of hemicellulose, cellulose, and lignin, which are chemically covalently bound together [55]. Hard carbon derived from biomass exhibits high reversible capacity, excellent performance, and low platform potential for Na^+^ ion storage in contrast to soft carbon with short-range order/long-range disorder graphene layers and graphite with long-range ordered graphene layers. Because of these properties, biomass-derived hard carbon is considered the most competitive anode material for commercial SIBs. The pyrolysis under 1000 °C is considered as the optimal temperature range for the formation of a well-developed porous structure and heteroatom doping. In pyrolysis at this temperature, volatiles such as hydrocarbons, H_2_O, CH_4_, CO_2_, CO, NH_3_, etc., are released. When the temperature exceeds 1000 °C the removal of some heteroatoms results in the arrangement of carbon layers to form nanovoids. Consequently, a temperature higher than 1000 °C is considered optimized for the preparation of high-performance BCMs [42].

The key benefit of the BCMs is that they maintain their natural microstructure after carbonization. As shown in Figure 3b, wood is a porous and fibrous structural tissue, which possesses a typical hierarchically porous structure, including micro-/meso-/macropores [56]. The hierarchical porous structure retained after carbonization can offer defective sites for Na^+^ ions storage, and shorten the solid-phase diffusion distance of Na^+^ ions. In addition, the native porous structure of BCMs can be constructed by traditional activation methods, i.e., physical activation and chemical activation [49]. Moreover, many biomass sources contain naturally enriched inorganic minerals [57]. During carbonization, these minerals can act as in situ templates; subsequent acid washing removes them and generates additional pores, thereby increasing the number of active sites available for Na^+^ storage.

While high-surface-area porous carbons were traditionally avoided due to concerns over excessive SEI formation and low initial Coulombic efficiency, they have recently emerged as a focal point for high-rate sodium storage [58]. Contemporary research now prioritizes the engineering of hierarchical pore structures and heteroatom doping to balance rapid ion diffusion kinetics with stable interfacial chemistry.

Lv et al. carbonized the mixture of peanut shells and KOH (7 wt%) at 600 °C to prepare porous carbon [59]. The porous carbon shows a finer porous structure with an increased percentage of micropores (<2 nm, 92.2%) and a larger specific surface area of 706.1 m^2^ g^−1^. The prepared porous carbon exhibits a capacity of 193 mAh g^−1^ at 0.25 A g^−1^ and a capacity of 130 mAh g^−1^ at 1 A g^−1^, superior to the carbon materials without activation. To make seashore-derived porous carbon with the adjusted surface area and pore structure, Senthil et al. [60] heated the carbon to 450 °C and activated it with KOH at 750 °C in a 1:3 ratio. The prepared porous carbon delivers remarkable reversible capacities of 303 mAh g^−1^ at a current of 100 mA g^−1^ and a reasonable rate capability. The excellent sodium storage performance can be attributed to the interconnected and porous nature, with the role of nitrogen dopant in defect creation and charge stabilization favoring Na^+^ ion storage, diffusion, and electron transfer.

Wang et al. reported a universal fungi-enabled method with KOH activation at 800 °C for the preparation of porous carbon with an ultrahigh specific surface area of 3439 m^2^ g^−1^ through the direct growth of mushrooms toward the cotton seed hull. The hyphae can bio-pretreat various components of host plants to produce pores by secreting an ample amount of exoenzymes, enabling the enzymatic solubilization of the plant cell wall at the micrometer and nanometer scales. This synthesis strategy could provide a new ideal for preparing biomass-derived porous carbon with high surface area, which has potential for the application of sodium storage [61].

There are countless animals on earth that provide many biomass resources for preparing different kinds of BCMs. Compared with plants, animal-derived biomass contains much more complex compositions [43]. Animal by-products are naturally pre-organized organic/inorganic nanocomposite materials composed of collagen and minerals. Making the best use of these natural nanocomposites is a good choice to develop carbon anodes for SIBs [62].

Liu et al. [63] transformed shrimp skin into nitrogen-rich mesoporous carbon through pyrolysis, leveraging collagen and minerals to create high-performance anode materials for SIBs. This process yielded a material with reversible sodium storage capacity of 434.6 mAh g^−1^ with excellent durability and rate capability, demonstrating a sustainable strategy for high-performance energy storage material. The aforementioned hard carbon exhibits significant porosity, high nitrogen content, and substantial interlayer spacing, leading to a much higher reversible specific capacity compared to conventional hard carbon. 

**Figure 4:**
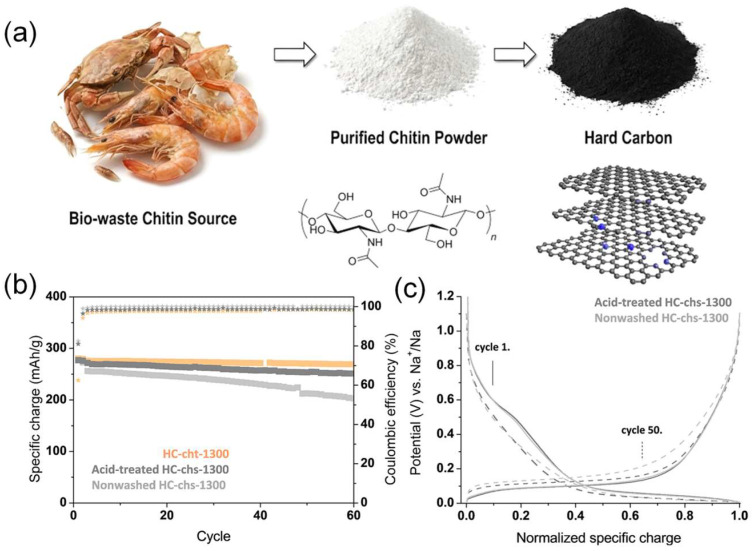
(**a**) Schematic illustration of the synthesis of nitrogen-doped hard carbons derived from bio-waste chitin. (**b**,**c**) Electrochemical performance of chitin and chitosan-based hard carbon anodes. Respective colored star signs represents Coulombic efficiency of the anodes. Reproduced from Ref. [65] with permission. Copyright 2019 American Chemical Society.

Chitin, abundant in the exoskeletons of insects, crustaceans, and fungi, is second only to cellulose among biopolymers. Its N-acetyl groups allow for direct pyrolysis, incorporating heteroatoms into carbocyclic rings to produce high-quality N-doped biocarbon materials (Figure 4). Chitin’s thermal stability varies by source but is generally higher than cellulose, with degradation starting around 280 °C. Major decomposition occurs between 300 and 450 °C, with a weight loss of 78.3% and a peak decomposition rate at 431 °C [43]. Hao et al. [64] made nitrogen-doped amorphous carbon nanofibers (NACFs) by directly heating chitin and using them as the anode material in SIBs. The NACF electrode achieved a high reversible capacity of 320.6 mAh g^−1^ with excellent rate capability and long cyclability. This superior performance is mainly due to the synergistic effects of the unique one-dimensional mesoporous nanofibers, which facilitate electron/electrolyte transmission, and the N-doped amorphous nanostructure, which enhances electrical conductivity and increases active sites. This inspires further exploration of advanced materials derived from renewable bio-waste (Table 2).

### 2.2. Hard Carbons from Polymer Precursors for Na-Ion Battery Anodes

In contrast to biomass-derived hard carbons, which often suffer from compositional randomness and batch-dependent differences, polymer-derived precursors offer a more controlled and reproducible pathway for hard carbon synthesis. Their well-defined molecular structures, predictable decomposition behavior, and adjustable functional groups allow for precise tuning of pore architecture, defect concentration, and interlayer spacing features that are essential for optimizing Na^+^ storage. As a result, polymer-based precursors serve as a complementary route to overcome the limitations of BCMs and enable more systematic engineering of hard carbon properties [71]. Polymeric precursors distinguish themselves from monomeric forms by possessing a preformed carbon backbone. This backbone can serve various purposes in terms of morphology and carbon structure, ultimately leading to positive implications for sodium-ion storage [72]. Typically, we prepare hard carbons from polymer precursors through direct sintering, microwave irradiation, or hydrothermal carbonization preprocessing; we generally form them at a temperature between 1000 and 1600 °C for several hours in a tubular furnace (TF) [73].

Cellulose, the most abundant organic polymer on Earth, is a sustainable source of carbon to use as a negative electrode for sodium-ion batteries. Simone et al. showed that the pyrolysis of a commercial cellulosic precursor led to hard carbons for which the microstructure depended on the final pyrolysis temperature [21]. A pyrolysis temperature of 1600 °C leads to hard carbon with extended nanodomains and smaller interplanar separations, which was completely contrary to the hard carbon samples achieved at 700 °C. A consistent trend was observed for pyrolysis temperatures between these two extremes. A capacity of 310 mAh g^−1^ was obtained for the sample prepared at 1600 °C, with major contributions from reductive pore filling by Na^+^ and lesser contributions from the intercalation mechanism. On the other hand, capacity contributions for the sample obtained at 700 °C are exclusively from intercalation. The results are consistent with the previously discussed structural evolution in hard carbons with increasing temperature [74]. Other biopolymers, such as lignin and hemicellulose, have not been studied often through direct pyrolysis for sodium-ion electrode applications, owing to unstable pyrolysis products from some of these precursors, but in combination with other polymers these have been shown to lead to useful carbon morphologies for the aforementioned application. A useful combination of natural and synthetic polymeric precursors was reported by Zhang et al. for the synthesis of functionally efficient battery-grade carbon [75]. They employed a bacterial cellulose/polypyrrole composite for the synthesis of core-sheath-structured porous hard CNFs. This carbon material delivered a capacity of 240 mAh g^−1^, with a remarkable cyclic and rate stability. The major contribution was shown to come from intercalation and surface-mediated storage [75]. A similar synergy of pitch/PAN was reported by Shi et al. for the synthesis of carbon fibers with composite hard and soft carbon features [76]. A capacity of 452 mAh g^−1^ was realized at a current of 20 mA g^−1^, which dropped to a value of 255 mAh g^−1^ at 200 mA g^−1^ after 200 cycles.

Polymer-/plastic waste-based precursors are abundant and sustainable materials for producing HC [12]. They often have a regular aromatic ring structure, which is conducive to the formation of highly ordered carbon crystals. The hard carbon materials prepared from polymers typically have high conductivity and good mechanical strength, a low surface area, a high pore volume, better structural stability, and a high capacity of more than 350 mAh g^−1^ [77]. Fan et al. employed a novel epoxy phenol resin as a precursor for the first time, producing hard carbon through the pyrolysis of epoxy phenol formaldehyde resin at 1800 °C. This hard carbon exhibited a reversible capacity of 480.3 mAh g^−1^ at 50 mA g^−1^ and a high initial Coulombic efficiency (ICE) of 84.6%. Impressively, even after 1000 cycles at 500 mA g^−1^, the capacity retention remained at 92%, indicating excellent long-term cycling stability. When selecting appropriate precursor materials, it is essential to thoroughly evaluate their advantages and disadvantages based on specific application requirements to achieve optimal HC [78].

The pyrolysis of easily processable polymers, such as polyacrylonitrile (PAN), polypyrrole, and polyaniline (PANI), has been effectively utilized for the synthesis of heteroatom-doped carbon structures, with their morphology being controlled through the processing of the precursor materials [79,80,81]. In this direction, hollow carbon nanowires have been reported by Cao et al. from the pyrolysis of hollow PANI precursors [36]. These hollow carbon nanowires delivered a capacity of 251 mAh g^−1^ with 82% retention over 400 charge–discharge cycles and equally good rate stability. In 2002, Thomas and Billaud demonstrated the high performance of PAN-derived hard carbon fibers for SIBs and reported a high capacity of 209 mAh g^−1^ [81]. The major contribution to the total capacity was observed to be from Na plating, as evident from the long plateau and small sloping regions. HC nanoparticles were synthesized by Liu et al. through the pyrolysis of PANI precursors [80]. The carbon obtained was N-doped and exhibited a remarkably high Na^+^ ion diffusion coefficient (10^−13^–10^−15^ cm^2^ s^−1^), which gave a high capacity of 298 mAh g^−1^ with high stability.

Hard carbons from organic polymer-based precursors mainly belong to organic resin precursors like polyacrylonitrile (PAN) [82], phenolic resin [83], epoxy resin [84], and conducting organic polymers such as polyaniline (PANI) [85]. Among these polymers, the resorcinol-formaldehyde resin (RF-resin) is an excellent precursor due to the ease of synthesis, availability, and high carbon yield. RF-resin is a thermosetting resin and forms a 3D network with more crosslinked structures after thermal treatment. The 3D network architecture can improve the battery’s long-cycle stability during charge and discharge processes. It makes the resinous precursors (RF and phenolic) an excellent choice for HC anodes in SIBs [83,86]. The resorcinol formaldehyde (RF)-resin as a precursor for mesoporous HCs via a hydrothermally controlled pyrolysis process was reported by Jin et al. [87]. The HC delivered a maximum reversible capacity of 310 mAh g^−1^ with the outstanding capacity retention of 96.7% after 100 cycles at a current density of 20 mAh g^−1^. 

The HC product obtained by pyrolyzing freeze-dried flakes of basil seed mucilage had a surface area of 40 m^2^ g^−1^. The natural gel-derived hard carbon (NGHC) has a reversible capacity of 214 mAh g^−1^ at a current density of 100 mA g^−1^, with long cycling stability up to 300 cycles. The NGHC shows an excellent rate performance of 95 mAh g^−1^ at 2 A g^−1^. It has a superior performance to that of commercially available HC. Some reports on biomass-derived hard carbons conclude that a greater amount of polymeric binding polymers, such as lignin, pectin, and hemicellulose, significantly improves the properties of HC for SIBs [88]. Mitlin et al. reported the synthesis of a carbon nanosheet framework obtained from peat moss, which contained lignin and hemicellulose in its composition [89]. They reported an impressive capacity of 298 mAh g^−1^ for the carbon material at a current density of 50 mA g^−1^, with a contribution of about 150 mAh g^−1^ from the intercalation path, mainly because of increased interplanar spacing. They found that a higher concentration of lignin and hemicellulose in the cellulosic biomass suppressed graphite nucleation. Suppressed nucleation produces carbon nanosheets with higher interplanar spacing. Suitable biomasses have also been pyrolyzed to achieve doping of heteroatoms in the carbon matrix. The influence of heteroatom doping for cases of non-biomass precursors has already been discussed above in previous sections.

**Figure 5:**
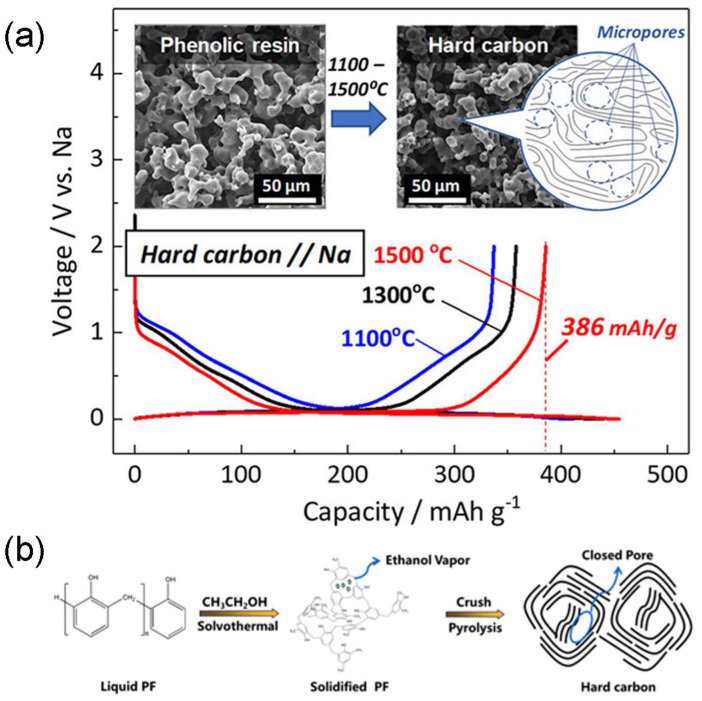
(**a**) The galvanostatic discharge/charge curves at 10 mA g^−1^ of phenolic resin-derived hard carbons carbonized at various temperatures. Reproduced from Ref. [90] Copyright 2020 American Chemical Society. (**b**) A schematic of the synthesis process of hard carbons using ethanol (EtOH) as a pore-forming agent. Reproduced from Ref. [91] with permission. Copyright 2019 American Chemical Society.

Komaba et al. [90] reported a series of microporous phenolic resin-derived hard carbons with ultrahigh reversible capacities. The hard carbons were synthesized by using a resin as the carbon precursor and an organic polymer as a pore-forming additive. Formalin and maleic acid as the crosslinker and catalyst, respectively, were also added for the preparation. After a series of curing, washing and carbonization processes, hard carbons were obtained. Increased carbonization temperatures led to a decrease in surface areas and an increase in pore sizes. Additionally, some of the open pores were transformed into closed ones by heat treatment above 1500 °C. As shown in Figure 5a, the resulting hard carbons at 1100, 1300 and 1500 °C were able to deliver reversible capacities of 337, 358 and 386 mAh g^−1^ with the corresponding ICEs of 82%, 81% and 85%, respectively. Hu et al. [91] also reported a resin-derived HC by tuning the closed pores for the highest sodium storage capacity. Phenol-formaldehyde resin (PF) was selected as a carbon precursor and ethanol (EtOH) was added as the pore-forming agent. The ratios of PF to EtOH were adjusted for the solvothermal process and the obtained intermediate products were further carbonized at different temperatures to fabricate the final HC materials with closed pores (Figure 5b). The optimal sample with a PF to EtOH ratio of 2:1 carbonized at 1400 °C had the best performance in a half-cell configuration, delivering a reversible capacity of 410 mAh g^−1^ at 0.1 A g^−1^ with a high ICE of 84%, and when evaluated in the full cell with NaNi_1/3_Fe_1/3_Mn_1/3_O_2_ as a cathode was able to reach a superior energy density of 300 Wh kg^−1^.

**Figure 6:**
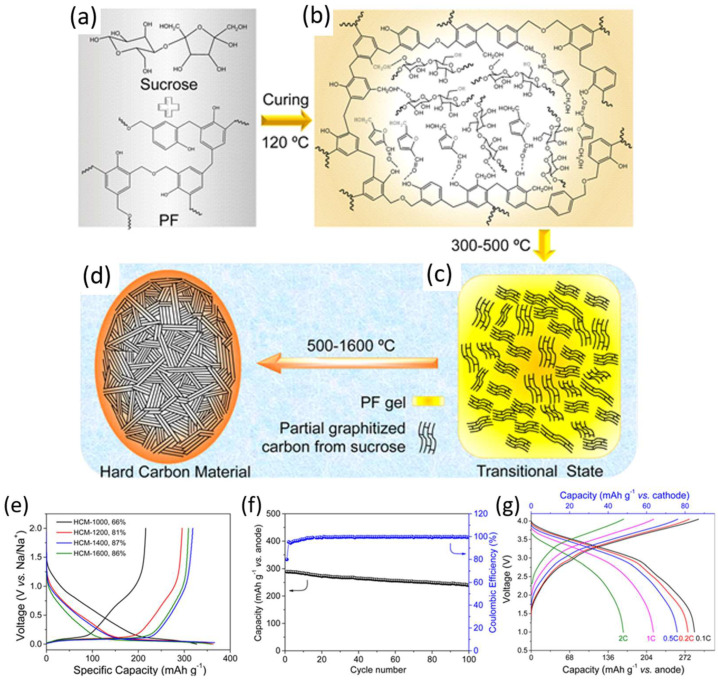
(**a**–**d**) Schematic illustration for HC preparation based on the mixture of sucrose and phenolic resin. Proposed mechanism for the formation of materials from sucrose/PF precursors leading to an extremely low surface area. (**e**) Voltage vs. capacity profile of HC, (**f**) full battery cycle performance at the rate of 0.2 C, and (**g**) full-cell charge/discharge rate curves. Reproduced from Ref. [92] with permission. Copyright 2017 American Chemical Society.

Yang et al. prepared a HC anode by carbonizing the mixture of abundant sucrose and phenolic resin (Figure 6a–d [92]). The polymer could cover the mixture surface, contributing to the low specific surface area of the as-prepared HC anode (1.54 m^2^ g^−1^), which is beneficial for suppressing excessive electrolyte decomposition and improving the ICE. In half-cell tests, the obtained carbon anode showed a reversible capacity of 319 mAh g^−1^ at 0.1 C and a good rate capability of 158 mAh g^−1^ at 1 C (Figure 6e). The heterostructure precursor strategy increased the ICE from 54% to 87%. When evaluated in a full-cell configuration using an O_3_-type Na_0.9_[Cu_0.22_Fe_0.30_Mn_0.48_]O_2_ cathode, the cell exhibited stable cycling over 100 cycles and delivered a capacity of 216 mAh g^−1^ at 1 C (calculated based on the anode active mass, Figure 6f,g). Similarly, Hu et al. [93] utilized a ball-milling method to mix pitch and phenolic resin in alcoholic solutions with different proportions, followed by carbonization under Ar atmosphere to obtain HC anodes. The electrochemical performance in half-cell configuration depended strongly on the pitch/phenolic resin ratio. An optimal reversible capacity of 284 mAh g^−1^ at 0.1 C was achieved with a pitch-to-phenolic resin ratio of 3:7, whereas improved rate capability was obtained at a 1:1 ratio. When assembled into a full sodium-ion cell with an O_3_-type Na_0.9_[Cu_0.22_Fe_0.30_Mn_0.48_]O_2_ cathode, the device delivered a reversible capacity of 240 mAh g^−1^ (based on the anode active mass) and an average operating voltage of 3.2 V, demonstrating its practical applicability. A hard–soft heterostructure carbon was made by Chou et al. [94] by mixing paper towel precursors and coal-tar pitch using a liquid-phase impregnation method. HC derived from the paper towel could provide active sites for high capacity, while soft carbon derived from coal-tar pitch prevented defects from decreasing ICE. The hard–soft heterostructure carbon (HC-P-1200) showed a much lower O/C ratio and a defective-less surface compared to the paper towel-derived HC (HC-W-1200). The HC-P-1200 anode showed a capacity of 293.2 mAh g^−1^, a plateau capacity of 224.9 mAh g^−1^ and good cycling stability with retention of 99% over 100 cycles at 20 mA g^−1^. The overall summary of the polymer-derived HC electrochemical performance in SIBs is presented in Table 3.

### 2.3. Reduced Graphene Oxides for Na-Ion Battery Anodes

Graphite is the go-to choice for Li-ion battery anodes, but it does not work well for Na-ion storage because the Na^+^ ion has a bigger radius, and it is difficult to intercalate into the tight graphite layers. When graphite is oxidized to graphite oxide (GrO), oxygen functional groups are introduced and the space between the layers increases. This makes it easier for sodium to be stored and greatly increases capacity [97]. Strong oxidation (like the Hummers’ method [98]) is usually used to make GrO, and then it is dispersed in a polar medium and then reduced (either thermally, chemically, or electrochemically) to partially restore conductivity, which makes reduced graphene oxide (rGO) [99]. Stand-alone rGO (without composite fillers) has become a promising sodium anode material because its structure and defect chemistry can be adjusted and tuned [100,101]. In contrast to hard carbons, reduced graphene oxide (rGO) consists of two-dimensional carbon sheets riddled with defects and residual oxygen functional groups, so sodium is stored predominantly by surface adsorption/pseudocapacitive processes at those defect sites, with only limited intercalation between its loosely restacked layers. Due to the structural and mechanistic differences, hard carbon typically exhibits a higher initial Coulombic efficiency (above 80%) and a near-zero-volt plateau, whereas rGO shows a more capacitive sloping voltage response and a lower initial efficiency due to greater irreversible Na^+^ uptake [102]. The following section will focus on rGO and its derivatives, highlighting how their unique graphene-based architecture can be implemented as potential SIB anodes.

**Figure 7:**
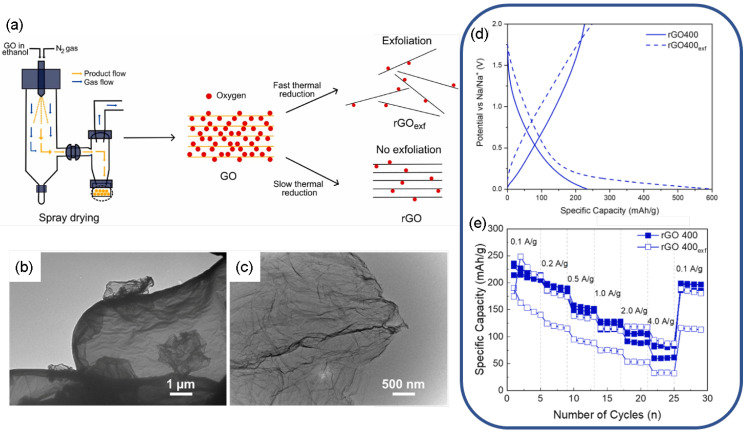
(**a**) Experimental protocol for the spray drying and modified thermal reduction in GO; (**b**,**c**) TEM images of the rGO400 and exfoliated rGO400 material, respectively; (**d**,**e**) electrochemical performance differences between exfoliated and non-exfoliated rGO obtained at 400 °C. Reproduced from Ref. [104]. Copyright 2025 Elsevier. Reprinted with permission under Creative Commons Attribution license [CC BY 4.0].

One of the key challenges with rGO anodes is that they usually exhibit a developed porosity and large surface area. As in practical applications, batteries are constrained more by their volume; a limitation with rGO-based anodes lies with their low bulk density due to the loosely stacked graphene nanosheets and large interparticle voids. Therefore, even when the gravimetric capacity (mAh g^−1^) is high, after electrode packing/calendaring, lower values of volumetric energy density (Wh L^−1^) are achieved. While high surface area provides many Na^+^ adsorption sites, it also leads to excessive solid–electrolyte interphase (SEI) formation and large initial irreversible capacity loss [97,103]. Dudding et al. [104] proposed a method to alleviate this issue: spray drying GO to create microspheres, followed by gradual thermal reduction to minimize exfoliation and surface area expansion (Figure 7a–c). The resulting rGO with a “low surface area” was reduced at 400 °C and had a reversible Na^+^ desodiation capacity of about 216 mAh g^−1^ at 0.1 A g^−1^, with 85% capacity retention after 200 cycles. This was over a double reduction in initial capacity loss compared to normal high-surface-area rGO (Figure 7d). This shows how changing the morphology of rGO (to lower surface area and optimal oxygen functional groups) can make the initial Coulombic efficiency and cycling stability better.

Another approach to improve the Na-ion storage in rGO-based materials is their structure modification using heteroatom doping (often with nitrogen, sulfur, or phosphorus). Nitrogen doping is very popular because N atoms (which have a higher electronegativity) can attract Na^+^ and also make the intralayer space larger and add more defects. It is important to note, however, that the electrochemical contribution of nitrogen is strongly configuration-dependent. Pyridinic and pyrrolic nitrogen species are generally considered more effective for Na^+^ adsorption due to their ability to create localized electron-deficient active sites, whereas graphitic (quaternary) nitrogen mainly enhances electronic conductivity without significantly strengthening ion adsorption. Multiple studies from both the battery and supercapacitor fields have shown that Na^+^ binds much more strongly to graphene near oxygen/N functional groups than to the pristine hexagonal C sp^2^ graphene structure. Shamim et al. [105] discovered through DFT calculations that the adsorption energy of Na^+^ near an epoxide group on N-doped rGO was approximately four times greater than that on pristine graphene, and that the presence of epoxides could enhance theoretical capacities by 2.5 to 3.5 times compared to a pure carbon lattice. Nitrogen dopants also improve material conductivity. During the experiments, free-standing N-doped rGO aerogels have performed much better compared to the undoped counterparts. Zhang et al. [106] described a 3D N-doped rGO aerogel anode that exhibits a high reversible capacity of about 288 mAh g^−1^ after 200 cycles at 0.1 A g^−1^. This N-rGO aerogel also had great rate capability, keeping ~152 mAh g^−1^ capacity even at a high current density of 5 A g^−1^. The improved performance was due to the 3D porous network, with favorable-sized channels for ion mobility, and the nitrogen-induced defects, which facilitated Na^+^ ion adsorption. Heteroatom-doped rGOs generally have higher specific capacities and better rate behavior than undoped graphene-based materials because they introduce more active sites and decrease the graphitic order to make room for Na^+^ ions [107,108].

## 3. Carbon-Based Composites for Next-Generation Na-Ion Battery Anodes

Transition metal compounds are another type of high-capacity anode materials, including transition metal oxides, sulfides, selenides, and borides, because transition metals usually possess various oxidation states. We can divide these transition metal compounds into two main categories. The first category is the compounds that only undergo conversion reactions, such as compounds of Ni, Co, Cu, Fe, Mn, Zn, and Mo. The other category are the compounds that can store Na^+^ ions through conversions and further alloying reactions. Such alloying reactions have the potential to provide additional specific capacity. This category includes the compounds of Sb, Bi, Ge, Sn, and Pb. Transition metal compounds suffer from low conductivity, serious particle aggregation, limited ionic diffusion rate, and volume expansion. Their practical application value is still limited [109]. Researchers have extensively researched carbon materials for energy storage and conversion due to their superior electric conductivity, large specific surface area, low cost, and structural controllability [110,111]. A combination of transition metal compounds and carbonaceous species is a popular hybrid that could offer a wide range of possibilities for fabricating high-performance and long-lasting SIB anodes [112]. Adding various carbon scaffolds to transition metal compounds can not only improve their intrinsic low conductivity but also change their shape. This is good for improving the reaction kinetics, fast charging/discharging rate, and cycling stability of transition metal compounds and carbon composites [110,113]. However, despite the improvements offered by carbon scaffolds, carbon–metal composites still face significant challenges that limit cycling stability [6]. The large volume expansion and contraction associated with conversion and alloying reactions (e.g., in Sn, Sb, or MoS_2_ systems) can induce mechanical stress, particle pulverization, and loss of electrical contact, leading to rapid capacity decay during repeated cycling [114]. Moreover, weak interfacial bonding between active metal phases and carbon matrices can lead to detachment and structural degradation at high cycles, further reducing electrochemical performance. While carbon can partially buffer mechanical strain and improve electron transport, it cannot fully eliminate these issues, and structural damage and interfacial instability remain major obstacles for practical applications [115].

### 3.1. Hard Carbon-Based Composites with Transition Metal Compounds for Na-Ion Battery Anodes

Regarding anodes in SIBs, hard carbons combined with transition metal chalcogenide is a rapidly growing topic. Transition metal chalcogenides (TMCs), usually be denoted as MX_2_ (M = Ti, V, Nb, Mo, W, Re; X = S, Se, Te), are emerging energy storage technologies. TMCs usually have a higher theoretical capacity; for example, MoS_2_ is considered a promising anode for SIBs. Hard carbons can be applied to restrict the volume expansion of TMCs. Ji and Zhang et al. [116] constrained exfoliated MoSe_2_ inside carbon spheres with a diameter of 100 nm, building a hybrid structure. Polyvinyl pyrrolidone (PVP) was used as a carbon precursor which can easily be dissolved and chemically attached to the surface of MoSe_2_. The electrochemical results for carbon–MoSe_2_ composites in SIBs are better than for bulk MoSe_2_; after 120 cycles, the specific capacity retained was around 441 mAh g^−1^ at a current density of 1 Ag^−1^ and 348 mAh g^−1^ at 4 Ag^−1^.

Various oxides, including Fe_2_O_3_, Fe_3_O_4_, Co_3_O_4_, NiO, CuO, MoO_3_, Mn_3_O_4_, SnO_2_, and SnO, have been investigated as SIB anodes. Among them, Sn-based oxides such as SnO_2_ and SnO are attracting special attention owing to their high theoretical reversible capacity, moderate operating voltage, good performance, and low cost [117]. HCs can serve as conductive hosts to accommodate transition metal oxides to achieve a variety of applications [118].

Three-dimensional (3D) porous γ-Fe_2_O_3_@C nanocomposites were prepared by aerosol spray pyrolysis. The nanocomposite demonstrates high-rate capability and long-term cyclability when applied as an anode material for Na-ion batteries. The desirable electrode shows a reversible capacity of 740 mAh g^−1^ after 200 cycles at 200 mA g^−1^ and high-rate performance with a discharge/charge capacity of 317 mAh g^−1^ at 8000 mA g^−1^. More importantly, after 1400 cycles the reversible capacity of 358 mAh g^−1^ was observed even at a high current density of 2000 mA g^−1^. The 3D porous Fe_2_O_3_@C nanocomposite can be made easily in one pot, and it has good electrochemical performance. This makes it a promising anode material for rechargeable SIBs [119].

With a high theoretical specific capacity and unique, intrinsic self-healing mechanism, the Sn_4_P_3_ alloy has been intensively investigated as an anode material for SIBs. The literature describes an approach that uses puffed rice HC with a loose structure, operating within the temperature range of 800 to 1400 °C to firmly encase the alloy through a straightforward ball milling process. The structurally stable Sn_4_P_3_@HC composites improve the electrochemical performance of SIBs as compared to the pristine Sn_4_P_3_ alloy and HC. At 1000 °C the composites show an excellent reversible capacity of 430 mAh g^−1^ at 100 mA g^−1^ over 100 cycles, with an elevated rate capability of 260 mAh g^−1^ even at a current density of 3.0 Ag^−1^, and a high capacity of 312 mAh g^−1^ after 400 cycles at 1.0 Ag^−1^. This work not only demonstrates a superior design of the HC and alloy, which synergistically stabilizes SIBs’ electrochemical performance, but also provides a simple, efficient, and easy-to-scale coating method for active materials [120].

**Figure 8:**
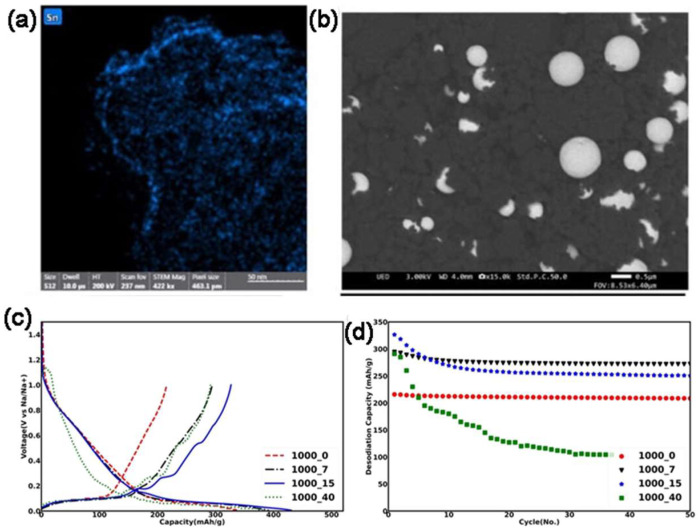
(**a**) TEM EDX image of the HC_Sn composite with 7% of tin; (**b**) SEM image of HC_Sn_1000_7 sample; (**c**) first GCD cycle of HC_Sn composites with various tin loading in a Na-ion half cell; (**d**) cycling performance at 50 mA g^−1^ of the HC_Sn composites and starting material. Reproduced from Ref. [121]. Copyright 2023 Wiley GmbH. Reprinted with permission under Creative Commons Attribution license [CC BY 4.0].

Another study focused on synthesizing Sn-doped HC composites to improve SIB performance. By doping HCs derived from Deregallera’s B187 and sucrose with different Sn contents, it was found that 7% Sn doping provides the best electrochemical performance. The Sn doping creates a synergistic effect by reducing the number of graphene layers in nano-graphitic domains, enhancing Na-ion access to nanopores. This structural change leads to increased first cycle Coulombic efficiency and capacity. Specifically, the 7% Sn-doped HC composite achieved a capacity of 285 mAh g^−1^, showing a 25% improvement over undoped carbon, outperforming the simple additive effect (Figure 8). This demonstrates that Sn doping is a cost-effective method for enhancing the performance of HC anodes in SIBs [121].

Apart from the metallic tin, mixtures and alloys of tin and antimony are also investigated for potential use in SIBs. Ding et al. [122] developed Sn/Sb@Sb_2_SnO_5_ encapsulated in N-doped porous carbon fibers (Sn/Sb@SSO@PCFs-N) as a composite anode material for SIBs. By using an electrostatic spinning technique, Sn and Sb nanocrystals were in situ generated around tin–antimony oxide (SSO), improving the composite’s electrical conductivity and Na^+^ ion transfer kinetics. The porous carbon fibers accommodate volume expansion during cycling, ensuring structural integrity. The composite delivers a high specific capacity of 450 mAh g^−1^ at 50 mA g^−1^ after 100 cycles and retains good rate performance, offering a capacity of 370 mAh g^−1^ even at 500 mA g^−1^. This method shows promise for developing cost-effective, high-capacity materials for next-generation SIBs.

### 3.2. rGO-Based Composites with Transition Metal Compounds for Na-Ion Battery Anodes

Even though conventional graphene-based anodes work well, combining rGO with transition metal compounds (oxides, sulfides, nitrides, etc.) can make both components work better together. As most oxides or sulfides have very high theoretical capacities when they react with sodium or form alloys, they have poor conductivity and big volume changes during electrochemical processes [108,123,124]. rGO is a great conductive scaffold and structural buffer for these materials. The rGO sheets are flexible enough to let transition metal compound nanoparticles expand while still keeping electrical contact, which stops particles from sticking together or coming apart during cycling. The surface of rGO can also evenly spread inorganic nanoparticles, often through in situ growth. This shortens the paths that Na^+^ ions can diffuse through and lowers the mechanical stress within the electrode [125].

**Figure 9:**
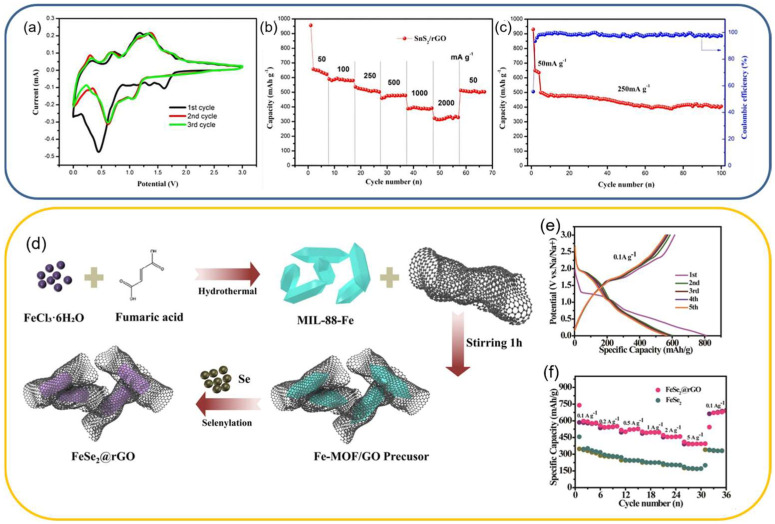
Electrochemical performance of SnS_2_/rGO composite: (**a**) CV profiles; (**b**) rate performance at the current range of 0.05 to 2 A g^−1^; (**c**) cyclic stability tests. Reproduced from Ref. [126]. Copyright 2018 Chen, Zhang, Zhang, Yu, Zheng, Ding, Li, Ming, Bengono, Chen and Tong. Reprinted with permission under Creative Commons Attribution license [CC BY 4.0]. (**d**) Experimental scheme of FeSe_2_/rGO composite fabrication; (**e**) charge–discharge profiles of the FeSe_2_/rGO; (**f**) rate performance of the FeSe_2_ and FeSe_2_/rGO composite. Reproduced from Ref. [129] with permission. Copyright 2021 Wiley GmbH.

For example, SnS_2_ nanosheets grown on rGO deliver a high initial capacity (~645 mAh g^−1^ at 0.05 A g^−1^) with far superior cycling stability than SnS_2_ alone [126]. The optimized amount of rGO nanosheets in the composite (15 wt%) lowers charge-transfer resistance and buffers the SnS_2_ nanostructures’ volume expansion, yielding 81% capacity retention after 100 cycles, which provides better results than a similar SnS_2_ composite with glucose-derived carbon (Figure 9).

Zhou et al. [127] combined another type of sulfide (antimony) with rGO to produce a 3D flower-like Sb_2_S_3_@rGO composite. A hydrothermal treatment of GO and potassium antimony tartrate with thioacetamide at 180 °C yielded a 5 µm flower structure with 50 nm thick nanosheet. The 3D flower-like Sb_2_S_3_@rGO composite provided a high reversible specific capacity of 545 mAh g^−1^ at 100 mA g^−1^ and maintained 75.4% of the initial capacity after 200 cycles. The sodium storage mechanism within the composite is governed by diffusion processes, but due to the rGO presence, the Na^+^ ions are transported rapidly within the electrode material.

Graphene-based material can also act as a conducting scaffold and as a supporting layer for inorganic nanostructure deposition. Yang et al. [128] used first amino-functionalized silica nanospheres to prepare spherical 3D GO structures, followed by a solvothermal growth of MoS_2_ on the rGO surface. Finally, HF etching was employed to remove silica particles. The MoS_2_/rGO features a 3D macroporous architecture with abundant channels and a reasonably developed specific surface area, demonstrating exceptional performance as an electrode for sodium-ion batteries. The MoS_2_/rGO composite exhibits a specific reversible capacity of 525 mAh g^−1^ at a current of 0.5 A g^−1^. Cycling stability was investigated at a high current load of 5 A g^−1^ for 3000 cycles, and the anode showed 37% of capacity retention. The electrochemical performance of 3D MoS_2_/rGO may stem from its distinctive macro-architecture, characterized by plentiful transport pathways, minimal volumetric changes during charge/discharge cycles, and high conductivity resulting from the strong interaction between MoS_2_ and rGO. The authors also claim that this strategy is likewise applicable to other transition metal sulfides.

A simple approach of combining the iron oxide particles with rGO aerogel was presented by Dong et al. [130]. Through a hydrothermal self-assembly of GO and Fe precursors (iron nitrate nonahydrate), followed by a freeze drying protocol, an rGO composite in the form of a sponge with Fe_2_O_3_ nanocubes of 140 nm in size was prepared. The rGO@Fe_2_O_3_ composite was investigated as an anode for SIBs and presented a low ICE of 51%, but afterwards, the specific capacity at a current density of 1 A g^−1^ achieved 327 mAh g^−1^ after 2000 charge–discharge cycles. The developed unique architecture of the composite enables spatial confinement to achieve excellent electrochemical performance, which can not only accommodate volume changes in sodiated Fe_2_O_3_ nanocubes, but also ensure favorable transport kinetics of electrons and Na^+^.

Similarly to the transition metal sulfides, the composites of rGO with selenides are also heavily investigated for SIB anodes. Zhang et al. [129] used a metal–organic framework (MOF) of MIL-88-Fe as a material backbone, which was then covered with GO nanosheets and combined with selenium powder under hydrothermal conditions (12 h, 150 °C, Figure 9d). The MOF-derived 3D conductive framework enhances both the material’s conductivity and the transport of sodium ions. Furthermore, the robust coating with rGO nanosheets mitigates the volume expansion of FeSe_2_. With the developed specific surface area and pore volume, the electrochemical processes at the electrode/electrolyte interface are facilitated. Leveraging the aforementioned advantages, the FeSe_2_@rGO composite demonstrates a substantial reversible capacity of 350 mAh g^−1^ after 600 cycles at 5000 mA g^−1^, along with exceptional rate performance (Figure 9e,f).

Table 4 summarizes recent rGO and rGO-based composite anodes with their electrochemical performance as anodes for SIBs. The presented materials highlight that rGO conductivity and 2D continuous robust structure coupled with nanoscale design of the active phase provide a significant electrochemical performance enhancement, enabling efficient electron/ion transport and mitigating structural degradation.

## 4. Conclusions and Outlook

Carbon-based materials, especially hard carbon and reduced graphene oxide, show promising potential as anodes for sodium-ion batteries due to their structure, chemical stability, tunability and abundance. In recent years, extensive research has deepened our understanding of how the microstructure of carbon affects Na^+^ storage. In hard carbon, sodium is mostly stored by filling pores and the accumulation at voltages below 0.1 V. At higher voltages, it is also stored by adsorbing at defects and edges. When designed correctly, this dual-region storage, which shows up as characteristic sloping and plateau voltage profiles, has a high theoretical capacity and good energy density.

Recent progress in hard carbon anodes for SIBs made from biomass has shown that it is possible to change the porosity, interlayer spacing, and heteroatom content by carefully choosing the precursor and pyrolysis parameters. Materials made from lignin, cellulose, chitin, or food or agricultural waste work very well and have initial Coulombic efficiencies of over 80%, especially when they are designed to reduce excessive surface area and promote closed-pore formation. Likewise, the polymer-derived HCs, such as those from formaldehyde resins, also offer a platform to precisely control morphology and surface chemistry. In optimized systems, they can achieve reversible capacities of over 350 mAh g^−1^ and long-term stability of over 1000 cycles.

Along with hard carbon, reduced graphene oxide (rGO) and its derivatives provide a 2D conductive framework with great surface accessibility and functional groups that can be utilized for further modification or increasing the charge storage of Na ions. When used as anodes, pure rGO is usually capacitive and has a lower initial Coulombic efficiency. However, strategies like nitrogen doping and pore engineering can greatly improve their performance. Composites made with rGO, especially those with high-capacity transition metal sulfides, selenides or oxides, have shown synergistic effects that combine the capacity of the active phase with the mechanical strength and conductivity of graphene. The literature provides numerous examples of how hybrid designs can make high-performance SIB anodes with exceptional durability.

However, even with the aforementioned improvements, there are still challenges to overcome. For hard carbons, it is still important to find a balance between high initial Coulombic efficiency, scalable synthesis, and keeping energy density through the plateau region. For graphene-based systems, reducing irreversible capacity loss and improving the bulk/tap density, which is essential for translating high gravimetric performance into competitive volumetric energy density, are key requirements for practical sodium-ion battery applications. Also, for composites, it will be very important to make sure that the dispersion is uniform, the interfaces are stable, and the synthesis is affordable. These limitations drift from the traditional concerns over LIB resource constraints: while carbon anodes avoid cobalt, lithium, and natural graphite entirely, inconsistent precursor purity in biomass routes and low volumetric metrics can still hinder cost-competitiveness versus optimized LFP cells in the near term.

The development of the carbon-based anodes for the Na-ion battery industry is also strictly related to combined *post mortem* and operando characterization. Implementation of the operando small-angle X-ray scattering (SAXS), operando XRD, and operando Raman spectroscopy measurements allows for simultaneous tracking of the structural changes in the materials when the battery operates. These highly sophisticated analytical techniques need to be coupled with computational methods, providing a verification of the theoretical simulations and models with experimental data, in order to achieve the best-performing anode materials.

Future research aims to incorporate multiscale design principles, integrating findings from operando studies and modeling with green chemistry and scalable processing. By taking into account various aspects like leveraging biomass valorization, polymer chemistry, and nanomaterials engineering, it will be possible to translate lab-scale innovation into real-world sodium-ion energy storage solutions. Emerging scalable routes such as flash Joule heating or hydrothermal pre-treatment of agricultural waste already demonstrate multi-ton potential while lowering energy consumption and carbon footprint. Machine learning-guided precursor screening and pore architecture optimization further accelerate discovery, targeting ICE > 90%, tap densities > 1 g cm^−3^, and full-cell energy densities > 200 Wh kg^−1^ at the pack level. Carbon-based anodes are still one of the most promising ways to make SIB technologies safe, efficient, and available to everyone around the world.

## Figures and Tables

**Table 1:** Evolution of sodium storage mechanism models in hard carbon.

Model/Period	Key Claim	Structural Characteristics	Potential-Dependent Evolution *	Description
Stevens & Dahn (2000) [35]	Insertion–adsorption	Turbostratic carbon layers with micropores	slope + plateau	Foundational model describing dual storage regions; simplified interpretation based on early structural understanding.
Cao et al. (2012) [36]	Adsorption–intercalation	Expanded pseudo-graphitic domains (d_002_ ≈ 0.37 nm)	slope → plateau	Adsorption dominates the slope, followed by proposed intercalation at lower potentials; intercalation contribution remains debated.
Bommier et al. (2015) [37]	Adsorption–insertion–filling	Defects + pseudo-graphitic domains + closed pores	slope → transition → plateau	Three-stage model covering the entire electrochemical profile; transitional framework linking surface processes and pore filling.
Zhang et al. (2016) [38]	Adsorption–insertion	Structure-dependent HC microstructure	slope + plateau	Mechanism depends strongly on material structure; emphasizes coexistence of adsorption and bulk storage processes.
Eren et al. (2025) [41]	Adsorption–accumulation–filling	Operando SAXS/WAXS/Raman; negligible d_002_ expansion	slope → early plateau → late plateau	Operando evidence suggests classical intercalation is negligible in most disordered HCs; sodium accumulation at pore surfaces and quasi-metallic clustering dominate plateau storage.

* slope + plateau means coexistence of two regions; slope → plateau indicates mechanistic progression with potential and sequential evolution.

**Table 2:** Summary of biomass-derived hard carbons synthesis protocols and their performance as anode materials for SIBs.

Biomass Source	Synthesis Method	Morphology	Specific Surface Area [SSA, m^2^ g^−1^]	Initial Coulombic Efficiency	Capacity Retention (mAh g^−1^)/(Number of Cycles) @Current Rate	Ref.
NACF chitin	carbonization 700 °C	nanofiber morphology	369.48	48.2%	320.6 (50) @50 mA g^−1^	[64]
Bagasse-derived hard carbon	carbonization 1000 °C	sheet structure (HC 1000)	92.3	73.1%	~60 (1000) @1000 mA g^−1^	[66]
Camphor wood residues	carbonization 1300 °C	porous morphology	3.74	82.8%	268 (200) @50 mA g^−1^	[67]
Tea tomenta	carbonization 1100 °C	Rod-like morphology	39.78	---	262 (100) @280 mA g^−1^	[68]
Cucumber stem	hydrothermal + carbonization 1000 °C	---	1988	38.2%	198 (500) @200 mA g^−1^	[69]
Chickpea husk	sonochemical impregnation + carbonization 1100 °C	honeycomb-like morphology	1599	41.3%	~320 (500) @20 mA g^−1^	[70]
Seaweed-derived carbon	carbonization + KOH activation 750 °C	sheet-like carbon structure	1641	21.4%	192 (500) @100 mA g^−1^	[60]
Cotton roll	carbonization 1300 °C	braided fibrous morphology and hollow structure	38	83.1%	~305 (100) @30 mA g^−1^	[28]

**Table 3:** Summary of polymer-derived hard carbons synthesis protocols and their performance as anode materials for SIBs.

Precursor	Synthesis Method	Electrolyte	ICE	Capacity Retention (mAh g^−1^)/(Number of Cycles) @Current Rate	Ref.
Phenol formaldehyde resin + sucrose	carbonization 1400 °C	0.8 M NaPF_6_/EC-DMC (1:1 *v*/*v*)	87.0%	287 (150) @0.1 C	[92]
Phenol- formaldehyde resin (PF)	solvothermal followed by carbonization	1 M NaPF_6/_EC-DMC (1:1 *v*/*v*)	84.0%	410 (45) @0.1 C	[91]
Pitch + paper towel	carbonization 1200–1400 °C	1 M NaPF_6/_EC-DMC (1:1 *v*/*v*)	94.1%	293.3 (100) @20 mA g^−1^	[94]
Polyvinylpyrrolidone (PVP)	carbonization 600 °C	1 M NaClO_4_/PC + 2% FEC	98.2%	386.4 (100) @20 mA g^−1^	[18]
Resorcinol-formaldehyde resin	carbonization 1300 °C	1 M NaClO_4_/EC-DEC (1:1 *v*/*v*)	75.5%	≈300 (150) @50 mA g^−1^	[95]
Pitch	carbonization 600 °C	1 m NaClO_4_/EC-DEC (1:1 *v*/*v*)	56.0%	≈200 (4000) @1000 mA g^−1^	[96]

**Table 4:** Summary of rGO-based materials and composites and their performance as anode materials for SIBs.

Anode Material	Synthesis Method	Reversible Capacity [mAh g^−1^] @Current Density	High-Rate Performance [mAh g^−1^] @Current Density	Capacity Retention (%)/(Number of Cycles) @Current Rate	Ref.
rGO400 (low-surface-area)	Spray drying + slow thermal reduction	216 @0.1 A g^−1^	105 @2 A g^−1^	85% (200) @1 A g^−1^	[104]
N-doped rGO aerogel	Hydrothermal self-assembly + N-doping	288 @0.1 A g^−1^	152 @5 A g^−1^	~90% (200) @0.1 A g^−1^	[105]
Functionalized graphene sheets (FGS)	Low temperature exfoliation and high temperature reduction	603 @0.05 A g^−1^	214 @10 A g^−1^	87% (2500) @0.5 A g^−1^	[131]
SnS_2_/rGO	In situ growth (reflux & solvothermal)	645 @0.05 A g^−1^	320 @2 A g^−1^	81.2% (100) @0.05 A g^−1^	[126]
Flower-like Sb_2_S_3_@rGO	Hydrothermal	545 @0.1 A g^−1^	392 @15 A g^−1^	75.4% (200) @1 A g^−1^	[127]
FeSe_2_/rGO	Hydrothermal	458 @0.5 A g^−1^	205 @75 A g^−1^	96.5% (6000) @5 A g^−1^	[132]
3D MoS_2_//GO	Hydrothermal growth on amino-functionalized SiO_2_ nanospheres	599 @0.05 A g^−1^	492 @2 A g^−1^	95.6% (100) @0.5 A g^−1^	[128]

## Data Availability

Data availability is not applicable to this article as no new data were created or analyzed in this study.
